# Fipronil-induced enantioselective developmental toxicity to zebrafish embryo-larvae involves changes in DNA methylation

**DOI:** 10.1038/s41598-017-02255-5

**Published:** 2017-05-23

**Authors:** Yi Qian, Cui Wang, Jinghua Wang, Xiaofeng Zhang, Zhiqiang Zhou, Meirong Zhao, Chensheng Lu

**Affiliations:** 1 Beijing Advanced Innovation Center for Food Nutrition and Human Health, Key Laboratory of Microbial Technology for Industrial Pollution Control of Zhejiang Province, College of Environment, Zhejiang University of Technology, Hangzhou, Zhejiang 310014 China; 2 College of Life Science, Taizhou University, Taizhou, Zhejiang 318000 China; 3 Beijing Advanced Innovation Center for Food Nutrition and Human Health, Department of Applied Chemistry, China Agricultural University, Beijing, 100193 China; 4 Department of Environmental Health, Harvard T.H. Chan School of Public Health, Landmark Center West, Boston, MA 02215 USA

## Abstract

Enantioselectivity in the aquatic toxicity of chiral pesticides has been widely investigated, while the molecular mechanisms remain unclear. Thus far, few studies has focused on genomic expression related to selective toxicity in chiral pesticide, nor on epigenetic changes, such as DNA methylation. Here, we used fipronil, a broad-spectrum insecticide, as a model chemical to probe its enantioselective toxicity in embryo development. Our results showed that S-(+)-fipronil caused severer developmental toxicity in embryos. The MeDIP-Seq analysis demonstrated that S-(+)-fipronil dysregulated a higher level of genomic DNA methylation than R-(−)-fipronil. Gene Ontology analysis revealed that S-(+)-fipronil caused more differentially methylated genes that are involved in developmental processes. Compared with R-(−)-fipronil, S-(+)-fipronil significantly disrupted 7 signaling pathways (i.e., mitogen-activated protein kinases, tight junctions, focal adhesion, transforming growth factor-β, vascular smooth muscle contraction, and the hedgehog and Wnt signaling pathways) by hyper-methylation of developmentally related genes, which further induced the downregulation of those genes. Together, these data suggest that differences in DNA methylation may partly explain the enantioselectivity of fipronil to zebrafish embryos. The application of epigenetics to investigate the enantioselective toxicity mechanism of chiral chemicals would provide a further understanding of their stereoselectivity biological effects.

## Introduction

Although chiral pesticides always share identical physical and chemical properties, they exert different biological and physiological effects on target and non-target species^1^. The use of chiral pesticides is currently widespread, and more than 40% of pesticides used in China are chiral^2^. Currently, the role of enantioselectivity and the environmental fate and health risk are generally recognized.

The evidence accumulated to date indicated that many chiral pesticides are toxic to aquatic organisms, including algae, small crustaceans, fish and other economically important animals. In our previous studies, the organochlorine pesticide acetofenate, fungicide metalaxyl and the synthetic pyrethroids exerted enantioselective developmental toxicity in zebrafish embryos and small crustaceans^3, 4^. For instance, the effect of (+)-acetofenate on the embryonic development of zebrafish was more pronounced than (−)-acetofenate and resulted in severer yolk sac edema and pericardial edema^3^.

In addition to the traditional aquatic toxicity endpoints, changes in mRNA expression levels are regarded as important biological responses to environmental contaminants. In recent years, researches on enantioselective aquatic toxicity of chiral chemicals have focused on a few genomic changes, such as the effects on estrogen receptors and interleukins. For instance, the enantioselective induction/suppression of estrogen-responsive genes or hypothalamic-pituitary-thyroid axis-related genes was investigated to uncover the mechanisms behind the selective toxicity of permethrin and metalaxyl in zebrafish embryo-larvae^4, 5^. However, most of the studies on the enantioselective developmental toxicity of chiral pesticides conducted in the past 20 years merely described the effects and did not determine the underlying mechanisms. To the best of our knowledge, few reports has discussed the potential mechanisms for the enantioselective developmental toxicity of chiral chemicals in zebrafish at the global epigenetic level, which affects the early development.

In recent decades, scientists have demonstrated the critical importance of epigenetic modifications in altering the expression of genes involved in development and homeostasis^6^. Epigenetic mechanisms, such as DNA methylation, histone modification and non-coding RNAs, would affect the structure of chromatin^7^. DNA methylation, the covalent addition of a methyl group to the 5^th^ carbon of cytosine, is a typical epigenetic mark involved in gene silencing and genome maintenance^8^. In vertebrates, such as zebrafish, DNA methylation is primarily observed on cytosine-guanine dinucleotide motifs (CpG). Several developmental stages of zebrafish embryogenesis have been well-characterized, which makes this species an extremely useful experimental model^9^. Additionally, changes in DNA methylation levels are highly dynamic during development^10–12^, resulting in tightly regulated gene expression^13^. Disorders in DNA methylation status are readily induced by external stimuli, including environmental changes and exposure to chemicals or pesticides^8, 14^. These DNA methylation alterations suppress certain cellular signaling pathways, leading to disorders, such as metabolic syndrome, altered development and even cancer^15, 16^. Despite the well-known effects of some pesticides on methylation status and organism development, the relationship between DNA methylation and developmental disorders of zebrafish embryo-larvae, following exposure to chiral pesticides has not been investigated.

Fipronil, a broad-spectrum n-phenylpyrazole insecticide that contains a sulfur chiral center, was introduced for commercial use in the United States in 1996. It has been banned or limited in some countries due to its high toxicity to bees and aquatic organisms. Fipronil possesses an asymmetric sulfoxide moiety and has two enantiomers, designated as S-(+)- and R-(−)-fipronil. The enantioselective toxicity of fipronil has been investigated in a variety of aquatic vertebrates^17^, including Japanese Medaka^18^, rainbow trout^19^ and fathead minnows^20^. Previous results suggested that the enantiomers of fipronil were easily separated and could subsequently be used to assess their individual effects experimentally. These features make fipronil an ideal model chiral pesticide to investigate the underlying mechanisms of its toxicity.

In the current study, we utilized a well-established genome-wide DNA methylation analysis strategy to determine the role of DNA methylation during developmental disorders of zebrafish embryo-larvae caused by the enantioselective toxicity of fipronil. We discovered a novel epigenetic mechanism underlying the enantioselective developmental toxicity to zebrafish embryo-larvae treated with R-(−)-fipronil or S-(+)-fipronil. Compared with R-(−)-fipronil, S-(+)-fipronil had significantly great acute toxicity and developmental toxicity, as S enantiomer increased global DNA methylation levels to a greater extent and disrupted seven development-related signaling pathways through hyper-methylation of genes involved in early zebrafish embryo-larvae development than its antipode. Taken together, our data reveal the importance of epigenetic evaluations for determining the enantioselective toxic effects of chiral pesticides on non-target aquatic species.

## Materials and Methods

### Chemicals and preparation of fipronil enantiomers

Fipronil (+, −) 5-amino-1-[2, 6-dichloro-4-(trifluoromethyl)-phenyl]-4- (trifluoromethylsulfinyl)-1-H-pyrazole-3-carbonitrile; 97.6% purity) was purchased from Sigma-Aldrich (USA). Fipronil enantiomers were separated using a Jasco LC-2000 series HPLC system (Jasco, Japan) according to an established method^21^. The resolved fractions were manually collected at the HPLC outlet, evaporated until dry, and dissolved in ethanol. Configurations of the enantiomers were determined based on the signals on a CD (circular dichroism) detector, and the concentrations were determined via an Agilent 6890N gas chromatograph as previously described^22^. The isolated enantiomers were more than 99% pure. All other chemicals or solvents used in the present study were HPLC or analytical grade.

Stock solutions of fipronil (+, −) were prepared in HPLC-grade 100% ethanol. A series of working stocks were made in 100% ethanol at 1,000 times the final concentration to allow for a 1:1000 dilution with the embryo medium (EM: 0.137 M NaCl, 5.4 mM KCl, 0.25 mM Na_2_HPO_4_, 0.44 mM KH_2_PO_4_, 1.3 mM CaCl_2_, 1.0 mM MgSO_4_ and 4.2 mM NaHCO_3_) and to create a series of test solutions with a final ethanol concentration of 0.1%.

### Zebrafish husbandry and Embryo collection

Adult AB strain zebrafish (*Danio rerio*) were maintained in a recirculating system according to standard husbandry procedures^23^ at 28 °C with a 14/10 (dark/light) photoperiod (lights were turned on at 8: 00 am). The fish were fed three-times per day with either the zebrafish diet (Zeigler, Aquatic Habitats, Apopka, FL, USA) or live Artemia (Jiahong Feed Co., China).

Zebrafish embryos were acquired from spawning adult fish with a sex ratio of 1:2 (female to male). Spawning was induced in the morning as the light was turned on. Subsequently, fertilized embryos without visible malformations or symptoms were collected, cleaned, and staged^22^. Three-hour post-fertilization (hpf) embryos were selected with a stereomicroscope (Nikon, Japan) according to their previously described developmental characteristics^24^.

### Embryo-larvae acute toxicity assays

Zebrafish embryos were exposed to 0, 100, 200, 400, or 800 μg/L fipronil (+, −) for 6–120 hpf based on the pretest results. Briefly, a single normal embryo was randomly distributed into each well of a 96-well plate, with 200 μL of control or fipronil solutions and half of the solution volume was renewed every 24 h. For each group of 32 embryos (n = 32), 4 replicates were performed. Plates were placed in a temperature-controlled incubator at 28 ± 1 °C with a 14/10 dark/light photoperiod. At 120 hpf, zebrafish larvae were anesthetized with ~0.02 g/mL MS222 (for imaging. pH = 7.0 ± 0.4). All the experimental protocol was approved by the Institutional Animal Care and Use Committee (IACUC) of Zhejiang University of Technology. All experiments were performed in accordance with relevant guidelines and regulations. The lengths from the anterior end of the mouth to the end of the caudal peduncle along the notochord of individual larvae were measured from digital micrographs.

### DNA sample preparation

Following incubation at 120 hpf, the zebrafish larvae from each group were collected and homogenized, and the genomic DNA was isolated using the DNeasy Blood & Tissue Kit (QIAGEN; Germany) according to the manufacturer’s instructions. The quality and concentration of genomic DNA were determined using a NanoDrop spectrophotometer (NanoDrop, USA). The integrity and quality of all DNA samples were tested. At least 2 μg DNA per sample with an A260/A280 ratio of 1.8–2.0 was used to confirm the genomic integrity by agarose gel electrophoresis (Supporting information Figure S1). Samples that met all criteria were used for the following MeDIP-Seq analysis.

### Methylated DNA immunoprecipitation and sequencing (MeDIP-Seq)

Genomic DNA was fragmented by sonication (Covaris, USA) into 150–500-bp fragments. The end of each DNA fragment was repaired and ligated to a 3′-A overhang using the NEXTflex™ Methyl End Repair and Adenylation Kit (Bioo Scientific, USA). Illumina sequencing adapters were ligated to the ends using the NEXTflex™ DNA Adapter or Barcode kit (Bioo Scientific). Double-stranded DNA was denatured, and DNA fragments were immunoprecipitated using 5-methylcytosine antibody beads (Diagenode, USA). The quality of immunoprecipitated fragments was validated by quantitative real-time polymerase chain reaction (qPCR). DNA fragments of 200–300 bp were excised from the gel and purified using MinElute Gel Extraction Kit (Qiagen). The extracted fragments were quantified using the Qubit™ dsDNA High Sensitivity Assay Kit (Invitrogen; USA) on an Agilent 2100 Analyzer (Agilent Technologies; USA). After qPCR analysis, the DNA libraries were sequenced (paired-end, 50-bp read length) using the Illumina HiSeq 2000 platform (Illumina). After the completion of a sequencing run, raw image files were processed with the Illumina Real-Time Analysis (RTA) software for base calling. Sequencing reads were deposited in the NCBI Short Read Archive (SRA) (Supporting information Figure S2a–g).

### Bioinformatics analysis

A Perl program was used to separate low-quality sequences from the raw sequencing data. The quality of each base was checked from the first base of each read. Once a low-quality base (quality <10) was identified, it was removed along with the following sequence. Paired-end reads with less than 30 bases remaining after trimming off the low-quality bases were removed. Bowtie (version 0.12.8) was used to map the remaining high-quality reads to the Ensembl zebrafish genome using the default mapping parameters^25^. We summarized the locations of unique reads in the reference genome.

After the alignment of high-quality reads to the reference genome, the Model-based Analysis of MeDIP-Seq (MACS) software^26^ was used to reveal peaks in the genome, and the locations of these peaks were noted. In addition, a Perl program was used to process the Bowtie mapping results and generate a profile of the relative peak expression. Then, edgeR^27^ was used to identify differentially expressed peaks between the two groups. The method used an empirical Bayes estimation and exact test based on the negative binomial distribution. Peaks with a P value ≤ 0.01 and expression ratio ≥2 or expression ratio ≤0.5 were considered significantly different between the two groups.

To clearly describe and compare the differentially expressed peaks, two databases, the Gene Ontology (GO) and Kyoto Encyclopedia of Genes and Genomes (KEGG) were used for functional annotation. GO terms were downloaded from Ensembl Biomart. Genes related to differentially expressed peaks were compared with the KEGG database (release 58)^28^ using BLASTX^29^ with an E value cut-off ≤1^−10^. Then, a Perl program was used to retrieve KO information from the blast results to associate genes with pathways.

### Real-time RT-PCR Analysis

Total RNA from zebrafish larvae was isolated with Trizol reagent according to the manufacturer’s instructions (Invitrogen, USA). Relative gene expression was measured using SYBR green mix (Promega, USA) on a qRT-PCR machine (Bio-Rad). Primer sequences for PCR reaction were as follows. BMP 7α, forward: 5′-GGTCGGCAGGACTGGATCAT-3′, reverse: 5′-ACCAGTGTCTGGACGAT AGC-3′; BMP 8, forward: 5′-TCGCTGGCTTCTCCATCCT-3′, reverse: 5′-GCC GTCCACTGCTATGATTT-3′; WNT 6, forward: 5′-GGTTATGGACCCTACC AGCA-3′, reverse: 5′-GGAACTGGAACTGGCACTCT-3′; protein kinase C δ, forward: 5′-TTTATTAACCCCAAGATGGAGCG-3′, reverse: 5′-AACTACA TTCAAGTAACCAG-3′; Rac 1, forward: 5′-ATGCAGGCCATCAAGTGTGTG-3′, reverse: 5′-CCGGTTTTCCATCTACCA TA-3′; actinin α2, forward: 5′-CTCGAGGCCGAGTACTGTATCAGTCGAA-3′, reverse: 5′-GGATCCAGTTAGGCTTTGTTCTCTTTATTTAGC-3′; myosin, forward: 5′-CTCAAGCGGGAGAACAAGAATC-3′, reverse: 5′-CTGAGGCTGAC CTGGTCTGTAA-3′; and GAPDH, forward: 5′-CGCTGGCATCTCCCTCAA-3′, reverse: 5′-TCAGCAACACGATGGCTGTAG-3′. GAPDH was applied as an internal control to determine the relative expression of those target genes.

### Statistical analyses

Spearman-Karber estimates ware applied to determine LC_50_ and EC_50_. All statistical analyses were performed in SPSS 16.0 (SPSS, Chicago, IL, USA), and data were compared using a one-way ANOVA with Fisher’s least significance difference (LSD) test to evaluate differences between the treatment groups. All values are presented as the mean ± standard error (SEM) and are considered significantly different when *p* < 0.05. All figures were created in Origin 8.0 (OriginLab, USA).

## Results

### Enantioselectivity in acute developmental toxicity of fipronil

**Figure 1:**
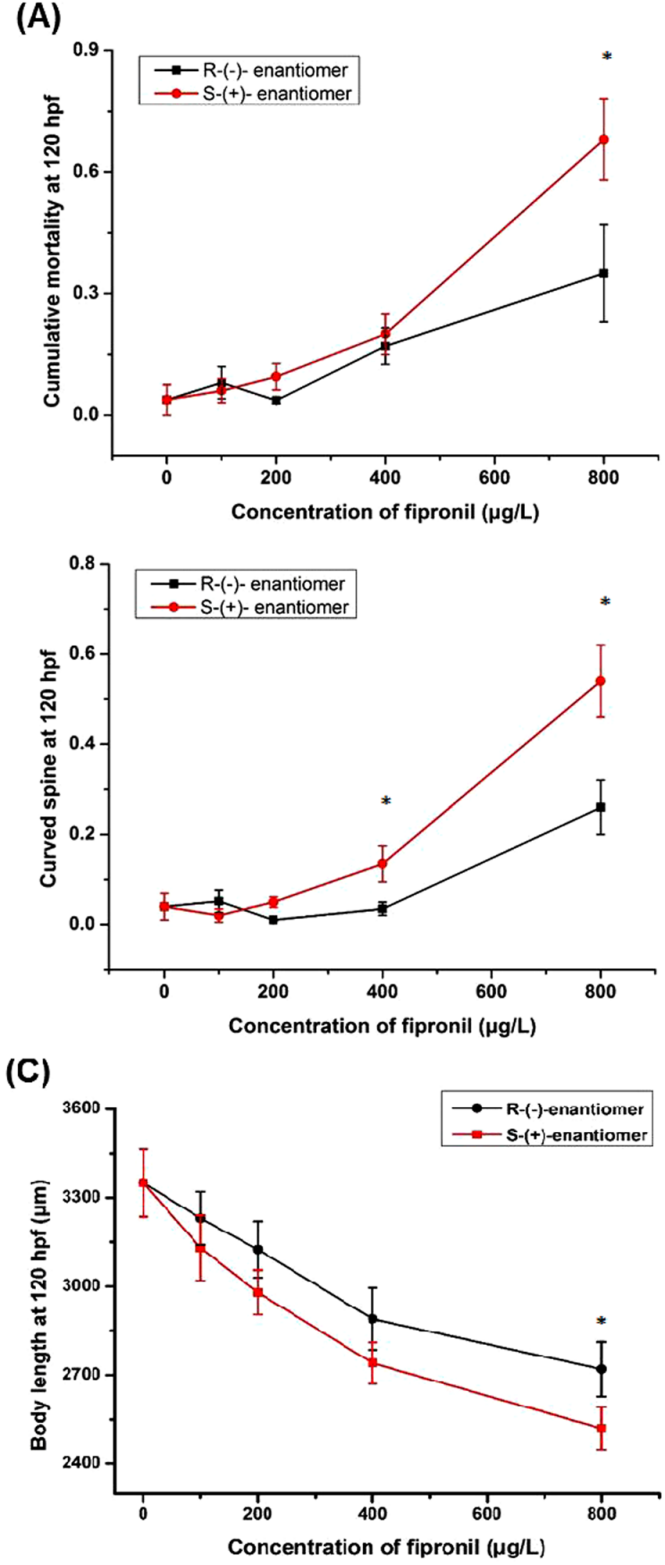
Enantioselective developmental toxicity of fipronil in zebrafish embryo-larvae. Zebrafish embryos were treated with fipronil (+, −) at different concentrations for 120 hpf (n = 32). The mortalities (**A**), curved spines (**B**) and body lengths (**C**) of embryo-larva were measured.

To test the enantioselective acute toxicity of fipronil, we first investigated the mortality of zebrafish embryo-larvae exposed to 100, 200, 400, or 800 μg/L of fipronil (+, −) from 6 to 120 hpf. The mortality and development of the zebrafish embryo-larvae were monitored every 12 h. As shown in Table 1 and Fig. 1A and B, the enantioselective effect was determined at the endpoints of mortality, body axis, and body length. The S-(+)-enantiomer was more toxic than its antipode; the LC_50_-_mortality_ (674 μg/L) and EC_50-curved body_ (776 μg/L) was calculated for the S-(+)-fipronil treated group. However, neither LC_50_ nor EC_50_ could be calculated in R-(−)-fipronil treated group at the same range of testing concentrations.

**Table 1:** Enantioselective effects of fipronil enantiomers (R, S) on the mortality and curved body axis of zebrafish larvae from 6 hpf to 120 hpf.

Concentration (μg/L)	Mortality^a^ (mean ± SEM)		Curved body axis^b^ (mean ± SEM)		_n_c
	S	R	S	R	
Control	0.033 ± 0.033	0.033 ± 0.033	0.04 ± 0.028	0.04 ± 0.028	96
100	0.062 ± 0.047	0.093 ± 0.048	0.021 ± 0.021	0.052 ± 0.01	96
200	0.094 ± 0.065	0.031 ± 0	0.042 ± 0.042	_**—**_^d^	96
400	0.195 ± 0.085	0.174 ± 0.061	0.134 ± 0.046	0.031 ± 0.018*	96
800	0.681 ± 0.11	0.396 ± 0.123*	0.537 ± 0.094	0.261 ± 0.068*	96
LC_50_/EC_50_ (μg/L)	674	**—**	776	**—**	

^a^Number of mortality of zebrafish larvae over the total number used for statistical analysis.

^b^Number of zebrafish larvae with curved body over the total number used for statistical analysis.

^c^Number of animals used for statistical analysis.

^d^No effects were detectable using the criteria of this study.

^*^Statistically significant difference between the two enantiomers exposed groups at a level of *p* < 0.05.

At 800 μg/L, exposure to the S-(+)-enantiomer resulted in a severer reduction of larvae body length than the *R*-(−)-enantiomer (Fig. 1C). At 120 hpf, 69 ± 3% of zebrafish embryos-larvae of the S-enantiomer had uninflated swim-bladders compared to 51 ± 5% for the R-enantiomer (data not shown). There was no difference in other exposure concentrations. Thus, the S-(+)-enantiomer had higher embryonic developmental toxicity.

### Global DNA methylation screen of the zebrafish larvae

Most studies have focused on enantioselective toxicity at the genetic level, neglecting its potential effects on epigenetic regulation. DNA methylation, associating with the repression of gene expression^30^, is the most sensitive epigenetic alteration, responding to subtle changes in the external environment^31^. Thus, we performed Methylated DNA Immunoprecipitation Sequencing (MeDIP-Seq), a high-throughput DNA methylation screening assay. Data of DNA methylation was sent to analysis the enantioselective changes between S- and R-enantiomer when DNA methylation in either group was significantly different from the control group. Up to 4.0 Gb of MeDIP-Seq data were generated. After the removal of adapter sequences, low quality reads, and contamination from raw reads, we obtained an average of 40 million sequenced reads (Supporting information Table S1). Among these reads, 43.67%–45.77% could be mapped. The number of uniquely mapped reads was 76.50%–77.46% of the total mapped reads.

The regions enriched in methyl-cytosine are known as methylation peaks. Methylation peaks are important parameters for analyzing global DNA methylation profiles^32, 33^. Thus, we analyzed the distribution of peaks across different genomic regions in each sample. As shown in Supporting information Figure S3, the frequency of methylation (7%) was significantly higher at promoters than downstream of transcription start sites (≤3 kb) in each group. In contrast, gene-body regions (intergenic, intron, exon regions) exhibited higher frequency of methylation, which was in agreement with previous studies^32, 34^. However, the most significant effects on gene expression were associated with altered DNA methylation in the promoter regions, which frequently contain CpG sites^30^. Thus, we analyzed the differentially methylated peaks in the promoter regions.

### Differential methylation status between S- and R-fipronil-treated groups

**Figure 2:**
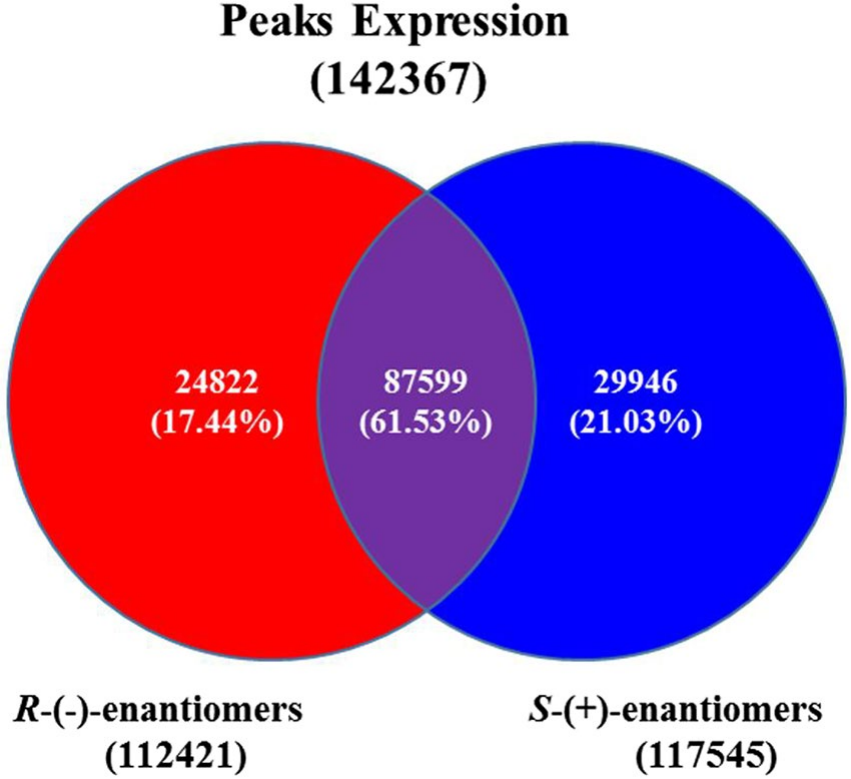
Regulation of global DNA methylation by fipronil (+, −). Zebrafish embryos were treated with 800 μg/L of the R-(−)-enantiomer or S-(+)-enantiomer for 120 hpf. Genomic DNA was extracted and analyzed by MeDIP-Seq.

We analyzed the differentially methylated regions in the genome of zebrafish embryos after exposure to S- or R-enantiomers of fipronil. As a result, 143,267 peaks (methylated regions in the genome) were detected in genes for the S- and R-enantiomer-treated groups, and 87,599 peaks were detected in both groups (Fig. 2, Table 2). Additionally, 29,946 peaks were detected only in the S-enantiomer group, which was approximately 20% more than in the R-enantiomer group. Thus, the S-enantiomer had a more profound effect on the regulation of global DNA methylation than the R-enantiomer. Moreover, to test whether S- or R-enantiomers affected DNA methylation differently, we examined the differentially expressed peaks between the two groups using edgeR. In agreement with the altered DNA methylation induced by the two enantiomers, the R-enantiomer increased methylation at 21,272 peaks, but 20% more peaks increased upon the treatment with the S-enantiomer (Fig. 2). Considering all the data, global DNA methylation could be increased by S- or R-enantiomers, but the S-enantiomer had a greater effect on DNA methylation than the R-enantiomer. This difference likely resulted in more severe global gene repression, and enhanced the acute toxicity in the zebrafish embryos.

**Table 2:** Statistics associated with expressed peaks of global DNA methylation in *R*-(−)-fipronil and *S*-(+)-fipronil.

Class	#	%
Total Peaks	192017	100
Expressed Peaks	142367	74.14
Expressed In *R*-(−)-enantiomers	112421	78.97
Expressed In *S*-(+)-enantiomers	117545	82.56
Expressed Both	87599	61.53
Expressed Only In *R*-(−)-enantiomers	24822	17.44
Expressed Only In *S*-(+)-enantiomers	29946	21.03
Differentially Expressed Peaks(p < = 0.01)& (ratio > = 2 or ratio < = 0.5)	Total #	47837
	Up #	21272
	Down #	26565

### Gene Ontology (GO) annotation and classification of the differentially methylated genes

**Figure 3:**
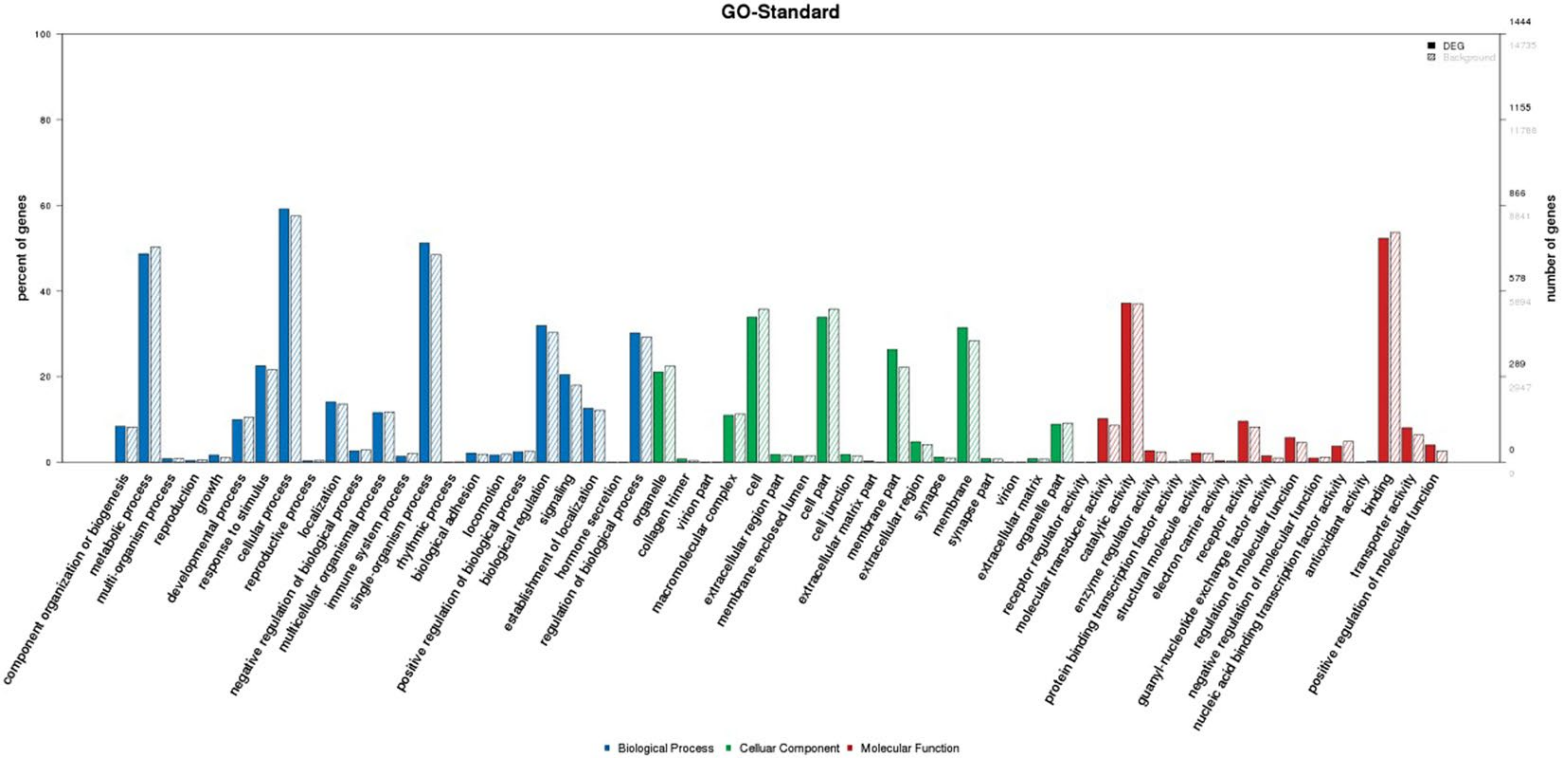
Functions of differentially methylated genes after exposure to fipronil (+, −) were annotated and classified using the Gene Ontology database.

GO annotation analysis was used to determine the potential roles of genes differentially methylated after treatment with the S- or R-enantiomers of fipronil. The GO analysis provides a controlled vocabulary of terms for describing gene products with annotation data (including “biological process,” “cellular component,” and “molecular function”). InterProScan software was used to annotate and classify the methylated genes according to their function. As illustrated in Fig. 3, large numbers of genes associated with the biological process category were differentially methylated in the groups treated with the R- and S-enantiomers. These genes were annotated with GO terms, such as “cellular process”, “single-organism process”, and “metabolic process”. Additionally, greater than 10% of differentially methylated genes (DMGs) were annotated with the GO term “developmental process” (Fig. 1B and C). Nearly 40% and 60% of DMGs had molecular functions with catalytic and binding activity, which is strongly associated with the embryonic development of vertebrates^35–37^. For the “cellular component” and “molecular function” categories, the majority of DMGs were associated with the cell membrane. These DMGs are predicted to have binding and catalytic activity, which is crucial for the maintenance of chromatin structure and embryonic development^35–37^. Thus, DNA methylation of these genes is potentially associated with the obvious developmental changes that occurred in the zebrafish embryos and the severe embryo mortality.

**Figure 4:**
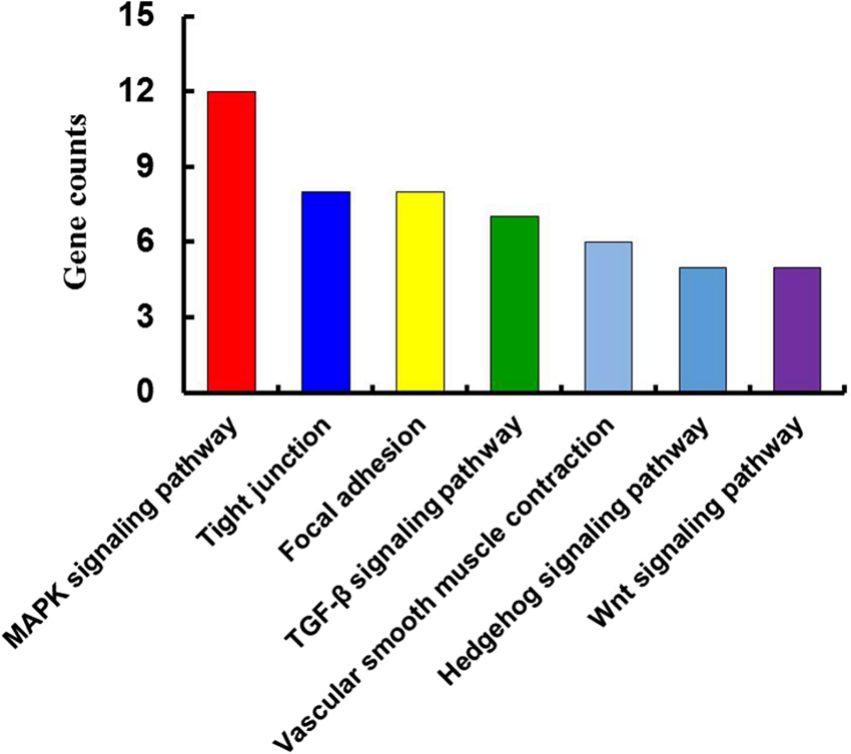
Pathways associated with more than 5 differentially methylated genes were analyzed with the KEGG database.

**Figure 5:**
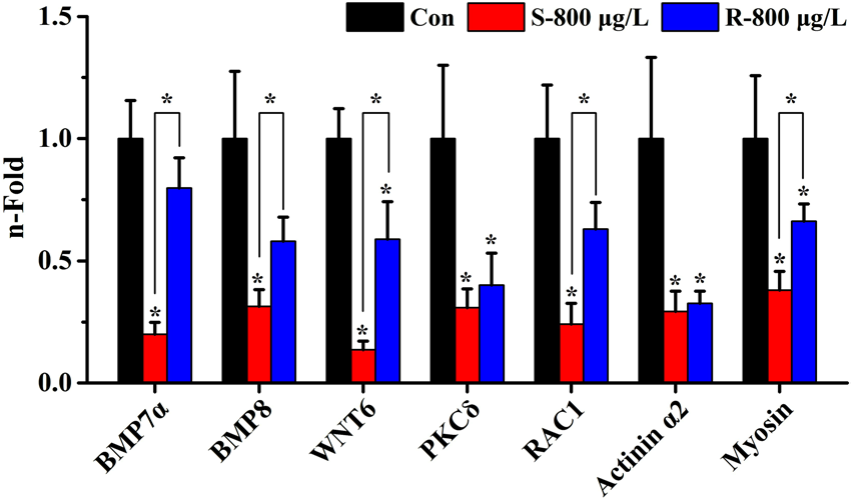
Regulation of seven hyper-methylated genes (BMP 7α, BMP 8, WNT 6, protein kinase C δ, Rac 1, actinin α2 and myosin) expression by S- fipronil or R- fipronil at the concentration of 800 μg/L.

To further explore the effects of the DMGs and the enantioselective effects of S- and R-(−)-fipronil on embryonic development and mortality, we used the Kyoto Encyclopedia of Genes and Genomes (KEGG) pathway database^38, 39^ to predict the pathways affected. Since S-(+)-fipronil exhibited more intense acute developmental toxicity, the hyper-methylated genes from the S-(+)-fipronil-treated group were compared to the R-(−)-fipronil-treated group (Fig. 1B and C). As a result, 22 pathways were identified that contained more than 5 hyper-methylated genes. Among those pathways, 7 (the mitogen-activated protein kinase (MAPK) signaling pathway, tight junction, focal adhesion, transforming growth factor β (TGF β), vascular smooth muscle contraction, and hedgehog and Wnt signaling pathways) were highly associated with developmental processes (Fig. 4). Among the hyper-methylated genes in the seven pathways (Table 3), we found that seven of the hyper-methylated genes (i.e., BMP 7α, BMP 8, WNT 6, protein kinase C δ, Rac 1, actinin α2 and myosin) were involved in two or more different pathways (Table 4), suggesting that these genes may serve a predominant role in development. Moreover, qRT-PCR was conducted to evaluate the expression of those seven hypermethylated genes. As a result, seven genes in S-fiponil exposed group were reduced to a greater extent than that in R-fiponil exposed group (Fig. 5), indicating that hypermethylation partially contributed to the downregulation of relevant genes, which are importantly involved in the biological processes.

**Table 3:** Observed differentially methylated genes (fold >5) in S-(+)-fipronil treated group involved in seven developmental associated pathways.

Gene	Fold
**MAPK signaling pathway (12 genes)**	
SH3-domain GRB2-like 1a	3.888
Arrestin 3, retinal (X-arrestin), like	5.832
Chemokine (C-X-C motif), receptor 4b	3.055
Fibroblast growth factor receptor 3	4.999
Heat shock cognate 70-kd protein, like; MCM5	3.611
Minichromosome maintenance deficient 5 (S. cerevisiae); heat shock cognate 70-kd protein; similar to heat shock protein 8	
Interleukin 2 receptor, beta	3.888
Kit receptor b	4.444
Similar to colony-stimulating factor 1 receptor a	6.666
Similar to development and differentiation enhancing factor 1	6.11
Similar to dynamin 1-like	4.444
V-erb-b2 erythroblastic leukemia viral oncogene homolog 3a	3.333
Similar to Centaurin-beta-1 (Cnt-b1) (ARFGAP with coiled-coil, ANK repeat and PH domain-containing protein 1) (ACAP1)	4.166
**Tight junction (8 genes)**	
Actinin alpha 2	3.055
Claudin k	3.611
Membrane protein, palmitoylated 5a	7.221
Myosin	4.444
Similar to B-regulatory subunit of protein phosphatase 2 A	4.444
Similar to Mitogen-activated protein kinase kinase kinase MLT	5.277
Similar to protein kinase C, delta	8.61
Protein phosphatase 2, regulatory subunit B, gamma a, ppp2r2ca	4.166
**Focal adhesion (8 genes)**	
Actinin alpha 2	3.055
Talin 2	3.333
Rac1	3.888
Myosin	4.444
Thrombospondin 5	4.999
Parvin, gamma	4.722
Baculoviral IAP repeat containing 7	3.888
Rap guanine nucleotide exchange factor (GEF) 1b	3.523
**TGF-β signaling pathway (7 genes)**	
Bone morphogenetic protein 7a	3.888
Bone morphogenetic protein 8	4.999
Inhibin, beta Ab	3.055
Nodal-related 1	5.555
Ribosomal protein S6 kinase b, polypeptide 1	4.444
Similar to activin receptor IIB thrombospondin 5	5.832
Transforming growth factor, beta 2	4.999
**Vascular smooth muscle contraction (6 genes)**	
Actin, alpha 2, smooth muscle, aorta	4.166
Adenosine A2a receptor a	4.444
Myosin	4.444
Similar to IP3 receptor associated cGMP kinase substrate A	4.556
Similar to alpha-1A adrenoreceptor	7.499
Similar to protein kinase C, delta	8.61
Actin, alpha 2, smooth muscle, aorta	4.166
**Wnt signaling pathway (5 genes)**	
WNT6	7.221
Prickle-like 1 (Drosophila) a	2.777
Rac1	3.888
Calcineurin-like EF-hand protein 1, chp1	2.777
Frizzled class receptor 6, Fzd6	2.777
**Hedgehog signaling pathway (5 genes)**	
GLI-Kruppel family member 1	3.333
Bone morphogenetic protein 7a	3.888
Bone morphogenetic protein 8	4.999
Low density lipoprotein-related protein 2	2.777
WNT6	7.221

**Table 4:** Differentially methylated genes involved in developmental-related pathways.

Gene	Pathway
Bone morphogenetic protein 7a	TGF-β signaling pathway
Bone morphogenetic protein 8	Hedgehog signaling pathway
WNT6	Vascular smooth muscle contraction
	Hedgehog signaling pathway
Similar to protein kinase C delt a	Tight junction
	Vascular smooth muscle contraction
Rac 1	Wnt signaling pathway
	Focal adhesion
Actinin α 2	Tight junction
myosin	Focal adhesion

## Discussion

The chiral insecticide, fipronil, had been extensively used on cotton, rice and corn crops as well as in commercial grass management and residential pest control for the past 20 years^19^. Although benefits have been derived from the use of fipronil, it may pose a risk to the aquatic environment^40^. Previous studies regarding the acute toxicity of fipronil to marine and freshwater non-target organisms revealed the enantioselectivity in the acute toxicity of fipronil is organ-specific^17^. Consistent with previous studies, our results indicated that zebrafish embryos exposed to fipronil (+, −) were significantly more vulnerable to S-(+)-fipronil than R-(−)-fipronil (Fig. 1). Similarly, Nillos *et al*. reported that S-(+)-fipronil was notably more cytotoxic to primary hepatocytes from rainbow trout than R-(−)-fipronil^19^. Taken together, data from the present study and previous studies indicated that S-(+)-fipronil was more toxic than R-(−)-fipronil to aquatic vertebrates, such as zebrafish, Japanese medaka, and rainbow trout.

DNA methylation is an epigenetic mechanism that plays a crucial role in regulating gene expression^41, 42^. A number of studies have shown that promoter methylation is directly associated with gene suppression; methylated promoters are unable to recruit RNA polymerase and other important transcription factors^43–45^. Numerous studies have investigated DNA methylation and the related toxicity^46, 47^. However, enantioselective patterns of DNA methylation have not been investigated regarding the toxicity of chiral pollutants. In the current study, both enantiomers of fipronil induced obvious changes in global DNA methylation. However, the S-(+)-enantiomer altered global DNA methylation more than the R-(−)-enantiomer, and pesticides with greater toxicity had more pronounced effects on DNA methylation^48, 49^.

Seven signaling pathways (MAPK, TGF-β, tight junction, focal adhesion, vascular smooth muscle contraction, hedgehog and Wnt) were predicted to play a role in developmental processes. Previous studies have demonstrated that MAPK signaling is required for the development of the subpallial telencephalon in zebrafish embryos^50^ and is an essential intermediate in vertebrate limb development^51^. During the early vertebrate development, tight junction signaling and the differentiation of the first epithelium in blastocysts is crucial for embryonic patterning and organization. Tight junctions provide epithelial layers with the capacity to confine and govern paracellular transport and, in the context of development, maintain the apico-basal polarity in membrane composition^52, 53^. Focal adhesion signaling is essential for embryonic development and plays a role in cell-extracellular matrix adhesion, cytoskeletal organization, polarity, migration, and survival during embryogenesis^54^. Similarly, TGF β and its superfamily members, the Nodal proteins, play important roles in mesendoderm induction and dorsoventral/anteroposterior patterning through their interaction with smad2 and smad3 during early vertebrate embryo development^55^. Additionally, the vascular smooth muscle contraction pathway is highly associated with the development of swimbladders in zebrafish larvae (swimbladders are highly vascularized organs)^48^. The cross talk between Hedgehog and Wnt signaling is necessary for the formation and organization of all three tissues layers of the swimbladder^49^. Additionally, previous studies have shown that the combination of Hedgehog and Wnt signaling was sufficient to induce myogenesis, and regenerate the zebrafish caudal fin in early developmental stages^56, 57^. Thus, our results indicated that the S-fipronil-dependent changes in DNA methylation likely induced the dysregulation of these signaling pathways, which was partly responsible for the significant developmental alterations.

Moreover, seven hyper-methylated genes (BMP 7α, BMP 8, WNT 6, PKC δ, Rac 1, actinin alpha 2 and myosin) were detected in two different developmentally related pathways, which suggests a significant role for these genes in zebrafish embryo development. The BMPs are growth factors that belong to the TGF superfamily that regulate multiple biological processes in development and morphogenesis^58, 59^. During early embryonic development, the activities of BMPs are crucial for dorso-ventral formation and the establishment of mesoderm-derived cell lineages^58^. A lack of functional BMPs in the embryos resulted in severe dorsalization, which adversely affected the specification of ventral and mesodermal cell fates^58, 60^. During organogenesis, BMPs regulate the morphogenesis of diverse organs. For example, reduced levels of BMPs induced defects in the formation of heart primordial cells and cardiac valves^60^. Additionally, BMPs regulate morphogenesis during vascular development by modulating the behavior of endothelial cells in vertebrates^61^. Protein kinase C δ is a vital regulator of vascular development, and its knockdown results in disorganized endothelial sprouting due to tip cell detachment from the stalk cell and a loss of polarization in the tip cells, which leads to altered angiogenic spouting during zebrafish development^62^. Similarly, Rac 1 is essential for embryonic development because its endothelial-specific deletion results in an early embryonic lethal vascular phenotype^63^. In Woo *et al*., downregulation the activity of Rac 1 in endodermal cells caused them to bypass the random migration phase and aberrantly contributed to mesodermal tissues and dysregulation of organogenesis in zebrafish^64^. Because these genes were hyper-methylated in the group treated with S-(+)-fipronil, they were significantly repressed, consistent with the finding described previously^42, 65^. This repression may be responsible for the more severe developmental problems in the S-(+)-fipronil-treated group. However, further investigation is supposed to elucidate the detailed molecular mechanisms underlying fipronil-mediated enantioselective alterations to DNA methylation status and its acute developmental toxicity to zebrafish.

In summary, the current study demonstrated that fipronil exerted enantioselective toxicity in zebrafish embryos through an epigenetic mechanism. Specifically, S-(+)-fipronil is more toxic than R-(−)-fipronil to zebrafish development, and this toxicity is reflected in differences in global DNA methylation, as well as in downregulation of genes involved in biological processes and molecular functions associated with development. Our study sheds new light on the enantioselective toxicity of chiral pesticides from the perspective of enantioselective epigenetic regulation.

## Electronic supplementary material

Supporting document.

## References

[1] Liu W, Gan J, Schlenk D, Jury WA (2005). Enantioselectivity in environmental safety of current chiral insecticides. Proceedings of the National Academy of Sciences of the United States of America.

[2] Zhang Q (2012). Enantioselective aquatic toxicity of current chiral pesticides. Journal of Environmental Monitoring: JEM.

[3] Zhao M, Liu W (2009). Enantioselectivity in the immunotoxicity of the insecticide acetofenate in an *in vitro* model. Environmental Toxicology and Chemistry/SETAC.

[4] Xu C (2008). Separation and aquatic toxicity of enantiomers of the pyrethroid insecticide lambda-cyhalothrin. Environmental Toxicology and Chemistry/SETAC.

[5] Jin Y (2009). Enantioselective induction of estrogen-responsive gene expression by permethrin enantiomers in embryo-larval zebrafish. Chemosphere.

[6] Bird A (2007). Perceptions of epigenetics. Nature.

[7] Qian Y (2015). Silver nanoparticle-induced hemoglobin decrease involves alteration of histone 3 methylation status. Biomaterials.

[8] Kamstra JH, Alestrom P, Kooter JM, Legler J (2015). Zebrafish as a model to study the role of DNA methylation in environmental toxicology. Environmental Science and Pollution Research International.

[9] Strahle U (2012). Zebrafish embryos as an alternative to animal experiments–a commentary on the definition of the onset of protected life stages in animal welfare regulations. Reproductive Toxicology.

[10] Lindeman LC (2010). Chromatin states of developmentally-regulated genes revealed by DNA and histone methylation patterns in zebrafish embryos. The International journal of Developmental Biology.

[11] Jiang L (2013). Sperm, but not oocyte, DNA methylome is inherited by zebrafish early embryos. Cell.

[12] Potok ME, Nix DA, Parnell TJ, Cairns BR (2013). Reprogramming the maternal zebrafish genome after fertilization to match the paternal methylation pattern. Cell.

[13] Aanes H (2011). Zebrafish mRNA sequencing deciphers novelties in transcriptome dynamics during maternal to zygotic transition. Genome Research.

[14] Collotta M, Bertazzi PA, Bollati V (2013). Epigenetics and pesticides. Toxicology.

[15] Wang J (2012). Nutrition, epigenetics, and metabolic syndrome. Antioxidants & Redox Signaling.

[16] Baylin SB, Jones PA (2011). A decade of exploring the cancer epigenome - biological and translational implications. Nature Reviews. Cancer.

[17] Overmyer JP (2007). Toxicity of fipronil and its enantiomers to marine and freshwater non-targets. Journal of Environmental Science and Health. Part. B, Pesticides, Food Contaminants, and Agricultural Wastes.

[18] Nillos MG, Lin K, Gan J, Bondarenko S, Schlenk D (2009). Enantioselectivity in fipronil aquatic toxicity and degradation. Environmental Toxicology and Chemistry/SETAC.

[19] Konwick BJ, Garrison AW, Black MC, Avants JK, Fisk AT (2006). Bioaccumulation, biotransformation, and metabolite formation of fipronil and chiral legacy pesticides in rainbow trout. Environmental Science & Technology.

[20] Baird S (2013). Enantioselective toxicity and bioaccumulation of fipronil in fathead minnows (Pimephales promelas) following water and sediment exposures. Environmental Toxicology and Chemistry/SETAC.

[21] Teicher HB, Kofoed-Hansen B, Jacobsen N (2003). Insecticidal activity of the enantiomers of fipronil. Pest Management Science.

[22] Jin M (2010). Dual enantioselective effect of the insecticide bifenthrin on locomotor behavior and development in embryonic-larval zebrafish. Environmental Toxicology and Chemistry/SETAC.

[23] Westerfield, M. *The Zebrafish Book: a Guide for the Laboratoryuse of Zebrafish (Danio rerio), third ed*. 267–272 (1995).

[24] Kimmel CB, Ballard WW, Kimmel SR, Ullmann B, Schilling TF (1995). Stages of embryonic development of the zebrafish. Developmental dynamics: an official publication of the American Association of Anatomists.

[25] Langmead B, Trapnell C, Pop M, Salzberg SL (2009). Ultrafast and memory-efficient alignment of short DNA sequences to the human genome. Genome Biology.

[26] Zhang Y (2008). Model-based analysis of ChIP-Seq (MACS). Genome Biology.

[27] Robinson MD, McCarthy DJ, Smyth G (2010). K. edgeR: a Bioconductor package for differential expression analysis of digital gene expression data. Bioinformatics.

[28] Kanehisa M (2006). From genomics to chemical genomics: new developments in KEGG. Nucleic Acids Research.

[29] Altschul SF (1997). Gapped BLAST and PSI-BLAST: a new generation of protein database search programs. Nucleic Acids Research.

[30] De Souza AP, Planello AC, Marques MR, De Carvalho DD, Line SR (2014). High-throughput DNA analysis shows the importance of methylation in the control of immune inflammatory gene transcription in chronic periodontitis. Clinical Epigenetics.

[31] Crews D, Gillette R, Miller-Crews I, Gore AC, Skinner MK (2014). Nature, nurture and epigenetics. Molecular and Cellular Endocrinology.

[32] Hu Y (2013). Comparison of the genome-wide DNA methylation profiles between fast-growing and slow-growing broilers. PloS One.

[33] Yan H (2010). Genome-wide mapping of cytosine methylation revealed dynamic DNA methylation patterns associated with genes and centromeres in rice. The Plant Journal: for Cell and Molecular Biology.

[34] Gim JA (2015). Genome-wide analysis of DNA methylation before-and after exercise in the thoroughbred horse with MeDIP-Seq. Molecules and Cells.

[35] Zhang YW, Arnosti DN (2011). Conserved catalytic and C-terminal regulatory domains of the C-terminal binding protein corepressor fine-tune the transcriptional response in development. Molecular and Cellular Biology.

[36] Chen Y (2014). Myeloid zinc-finger 1 (MZF-1) suppresses prostate tumor growth through enforcing ferroportin-conducted iron egress. Oncogene.

[37] Honarpour N (2014). F-box protein FBXL16 binds PP2A-B55alpha and regulates differentiation of embryonic stem cells along the FLK1+lineag. e. Molecular & Cellular Proteomics: MCP.

[38] Kanehisa M, Goto S (2000). KEGG: kyoto encyclopedia of genes and genomes. Nucleic Acids Research.

[39] Kanehisa M, Goto S, Sato Y, Furumichi M, Tanabe M (2012). KEGG for integration and interpretation of large-scale molecular data sets. Nucleic Acids Research.

[40] Wang C (2016). A metabolomic study of fipronil for the anxiety-like behavior in zebrafish larvae at environmentally relevant levels. Environmental Pollution.

[41] Jaenisch R, Bird A (2003). Epigenetic regulation of gene expression: how the genome integrates intrinsic and environmental signals. Nature Genetics.

[42] Watson RE, McKim JM, Cockerell GL, Goodman JI (2004). The value of DNA methylation analysis in basic, initial toxicity assessments. Toxicological Sciences: an official journal of the Society of Toxicology.

[43] Schulze I (2016). Increased DNA methylation of Dnmt3b-targets impairs leukemogenesis. Blood.

[44] Cho YD (2014). Epigenetic modifications and canonical wingless/int-1 class (WNT) signaling enable trans-differentiation of nonosteogenic cells into osteoblasts. The Journal of Biological Chemistry.

[45] Jiang X (2010). The imprinted gene PEG3 inhibits Wnt signaling and regulates glioma growth. The Journal of Biological Chemistry.

[46] Ozden S (2015). Assessment of global and gene-specific DNA methylation in rat liver and kidney in response to non-genotoxic carcinogen exposure. Toxicology and Applied Pharmacology.

[47] Hu J (2014). Effects of benzene and its metabolites on global DNA methylation in human normal hepatic l02 cells. Environmental Toxicology.

[48] Yafune A (2013). Global DNA methylation screening of liver in piperonyl butoxide-treated mice in a two-stage hepatocarcinogenesis model. Toxicology Letters.

[49] Lind L (2013). Global DNA hypermethylation is associated with high serum levels of persistent organic pollutants in an elderly population. Environment International.

[50] Shinya M, Koshida S, Sawada A, Kuroiwa A, Takeda H (2001). Fgf signalling through MAPK cascade is required for development of the subpallial telencephalon in zebrafish embryos. Development.

[51] Ma H, Blake T, Chitnis A, Liu P, Balla T (2009). Crucial role of phosphatidylinositol 4-kinase IIIalpha in development of zebrafish pectoral fin is linked to phosphoinositide 3-kinase and FGF signaling. Journal of Cell Science.

[52] Bhavwanti Sheth, J. E., Fay Thomas, Tom P. Fleming. In *Tight Junctions* 164–174 (Springer US, 2006).

[53] Fleming TP, Papenbrock T, Fesenko I, Hausen P, Sheth B (2000). Assembly of tight junctions during early vertebrate development. Seminars in Cell & Developmental Biology.

[54] Wu C (2007). Focal adhesion: a focal point in current cell biology and molecular medicine. Cell Adhesion & Migration.

[55] Jia S, Ren Z, Li X, Zheng Y, Meng A (2008). smad2 and smad3 are required for mesendoderm induction by transforming growth factor-beta/nodal signals in zebrafish. The Journal of Biological Chemistry.

[56] Yin A, Korzh S, Winata CL, Korzh V, Gong Z (2011). Wnt signaling is required for early development of zebrafish swimbladder. PloS One.

[57] Wehner D (2014). Wnt/beta-catenin signaling defines organizing centers that orchestrate growth and differentiation of the regenerating zebrafish caudal fin. Cell Reports.

[58] Kondo M (2007). Bone morphogenetic proteins in the early development of zebrafish. The FEBS journal.

[59] Sieber C, Kopf J, Hiepen C, Knaus P (2009). Recent advances in BMP receptor signaling. Cytokine & growth factor reviews.

[60] Kim JD, Kim J (2014). Alk3/Alk3b and Smad5 mediate BMP signaling during lymphatic development in zebrafish. Molecules and Cells.

[61] Wiley DM, Jin SW (2011). Bone Morphogenetic Protein functions as a context-dependent angiogenic cue in vertebrates. Seminars in Cell & Developmental Biology.

[62] Oubaha M (2012). Formation of a PKCzeta/beta-catenin complex in endothelial cells promotes angiopoietin-1-induced collective directional migration and angiogenic sprouting. Blood.

[63] Tan W (2008). An essential role for Rac1 in endothelial cell function and vascular development. FASEB journal: official publication of the Federation of American Societies for Experimental Biology.

[64] Woo S, Housley MP, Weiner OD, Stainier DY (2012). Nodal signaling regulates endodermal cell motility and actin dynamics via Rac1 and Prex1. The Journal of Cell Biology.

[65] Holliday R, Pugh JE (1975). DNA modification mechanisms and gene activity during development. Science.

