# Supplementary material for: Fipronil-induced enantioselective developmental toxicity to zebrafish embryo-larvae involves changes in DNA methylation
Source: Sci Rep. 2017 May 23;7:2284. doi: 10.1038/s41598-017-02255-5 (PMC5442102; doi:10.1038/s41598-017-02255-5)

***Supporting Information for***

**Fipronil-induced enantioselective developmental toxicity to zebrafish embryo-larvae involves changes in DNA methylation**

Yi Qian^1,2a^, Cui Wang^1a^, Jinghua Wang^1^, Xiaofeng Zhang^1^, Zhiqiang Zhou^3^, Meirong Zhao^1^* and Chensheng Lu^4^

1. *Beijing Advanced Innovation Center for Food Nutrition and Human Health, Key Laboratory of Microbial Technology for Industrial Pollution Control of Zhejiang Province, College of Environment, Zhejiang University of Technology, Hangzhou, Zhejiang, China*
2. *College of Life Science, Taizhou University, Taizhou, Zhejiang, 318000, China*
3. *Beijing Advanced Innovation Center for Food Nutrition and Human Health, Department of Applied Chemistry, China Agricultural University, Beijing, China*
4. *Department of Environmental Health, Harvard T.H. Chan School of Public Health, Landmark Center West, Boston, MA, 02215, USA*
5. ^a^ These authors contributed equally to this work.
6. ^*^To whom correspondence should be addressed. Phone: +86 571 8832 0265;

Fax: +86-571-88320265. Email: [zhaomr@zjut.edu.cn (M](mailto:zhaomr@zjut.edu.cn (M) Zhao)

**Supporting information, Table S1** The general information of reads alignment in the genomes of zebrafish larva exposed with R-(-)- enantiomer or S-(+)- enantiomer.

| **Class** | ***R*-(-)-enantiomer** | | ***S*-(+)-enantiomer** | |
| --- | --- | --- | --- | --- |
|  | **#** | **%** | **#** | **%** |
| **Total Reads** | 40285098 |  | 39741652 |  |
| **Total Mapped Reads** | 17591926 | 43.67 | 18190200 | 45.77 |
| **Total Mapped Uniquely Reads** | 13626800 | 77.46 | 13916178 | 76.50 |

**Supporting information, Figure S1** Quality control of Zebrafish embryo-larva genome DNA through DNA gel electrophoresis.


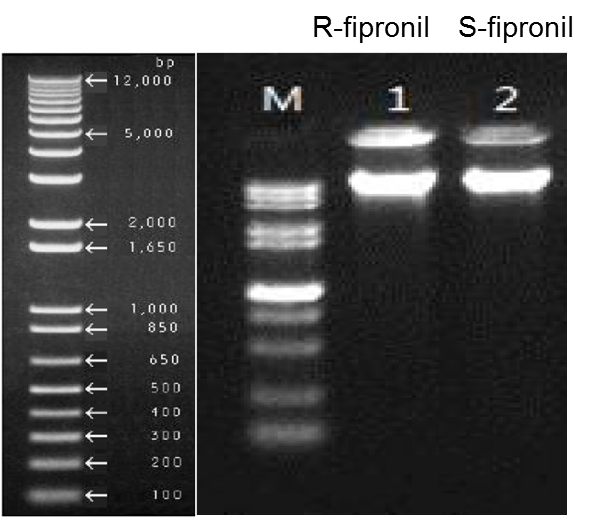


**Supporting information, Figure S2 a-g** Quality control of genome DNA Library via High Sensitivity DNA Assay.

**a
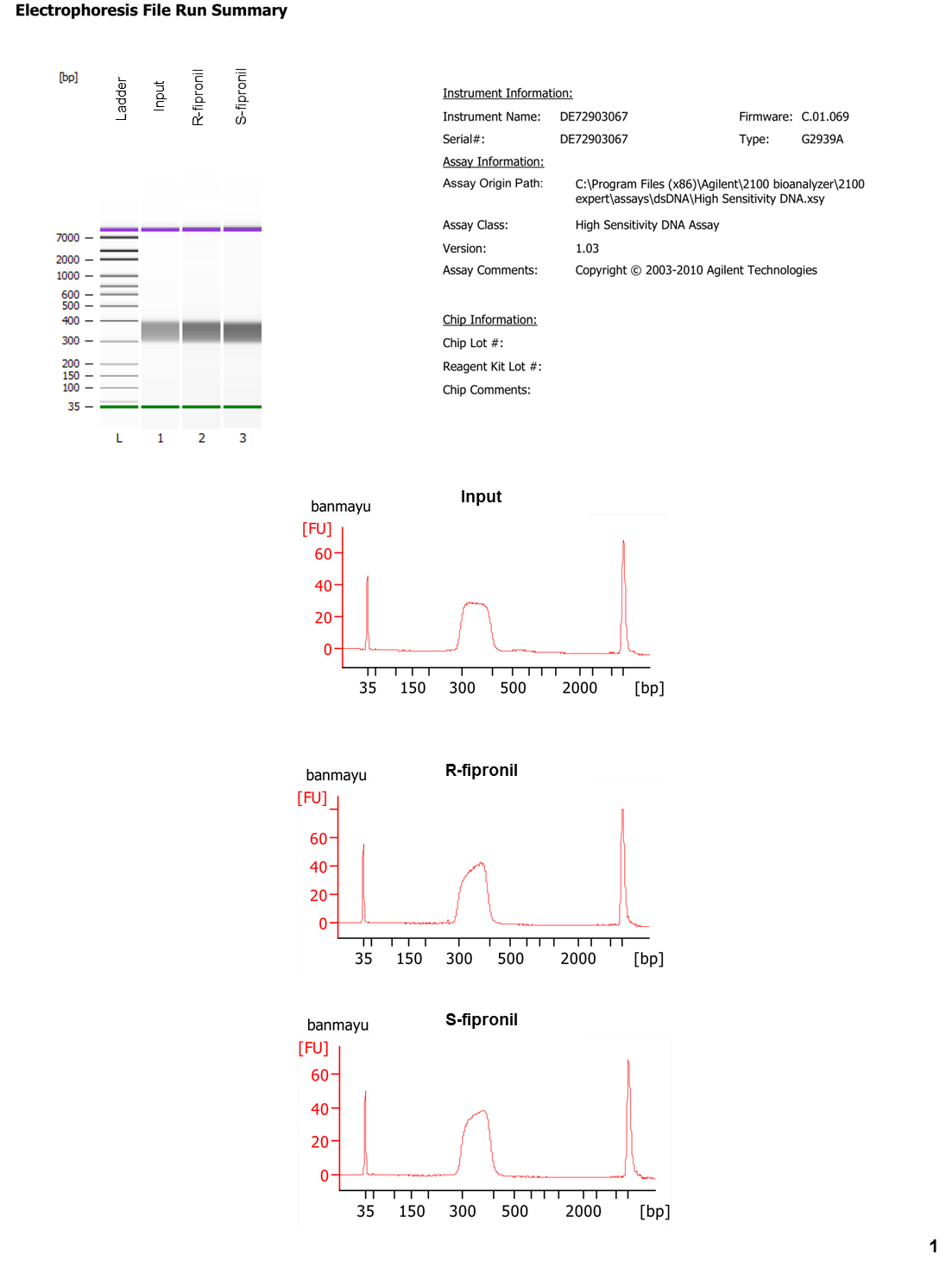
**

**b
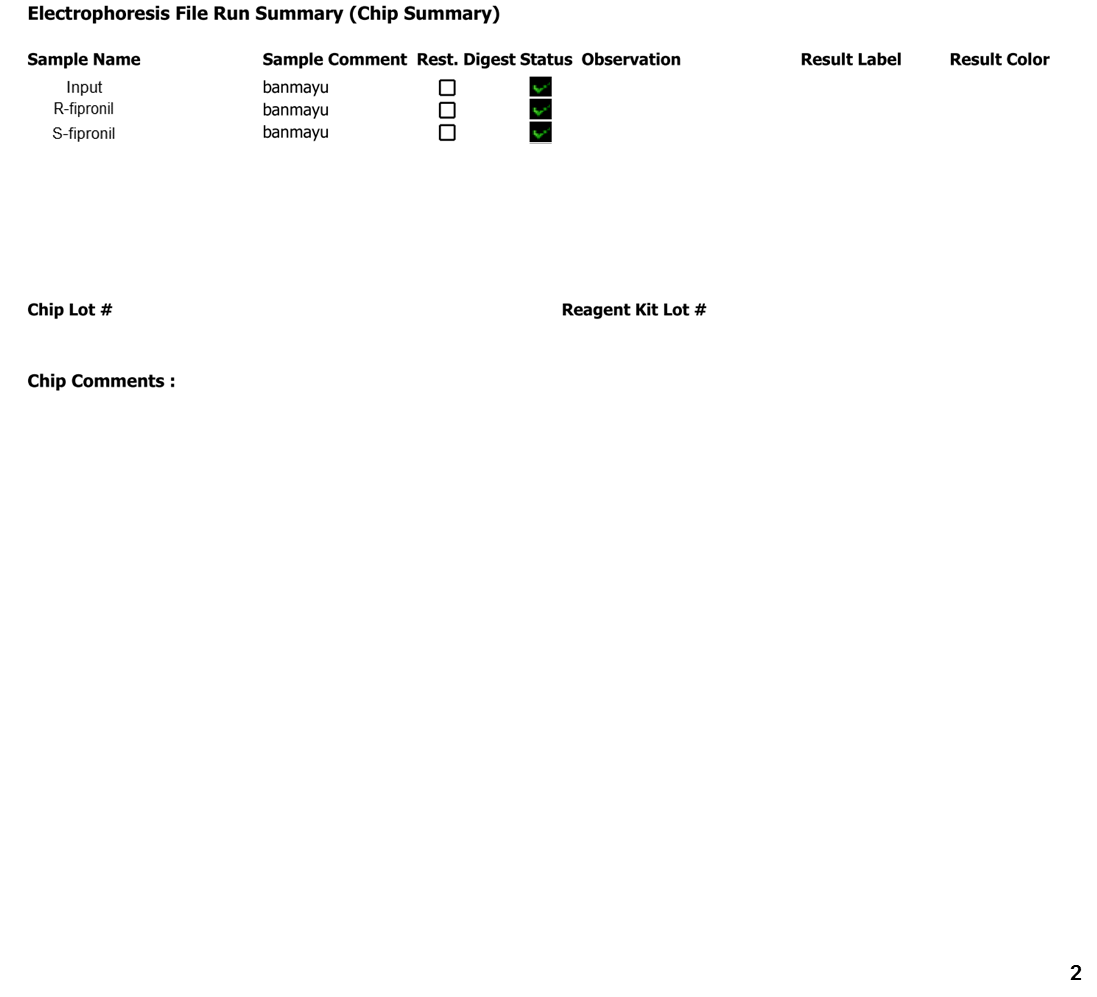
**

**c
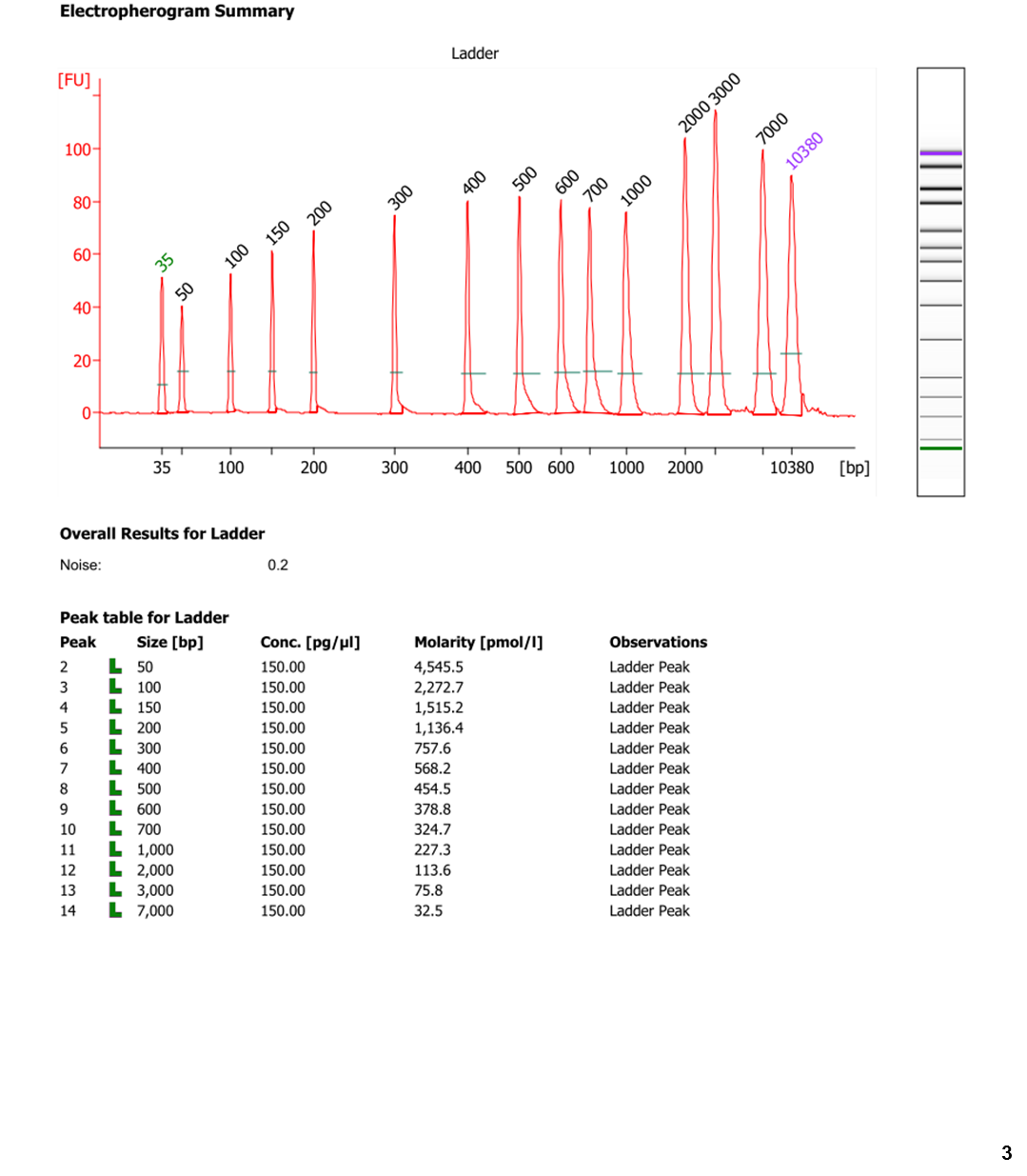
**

**d
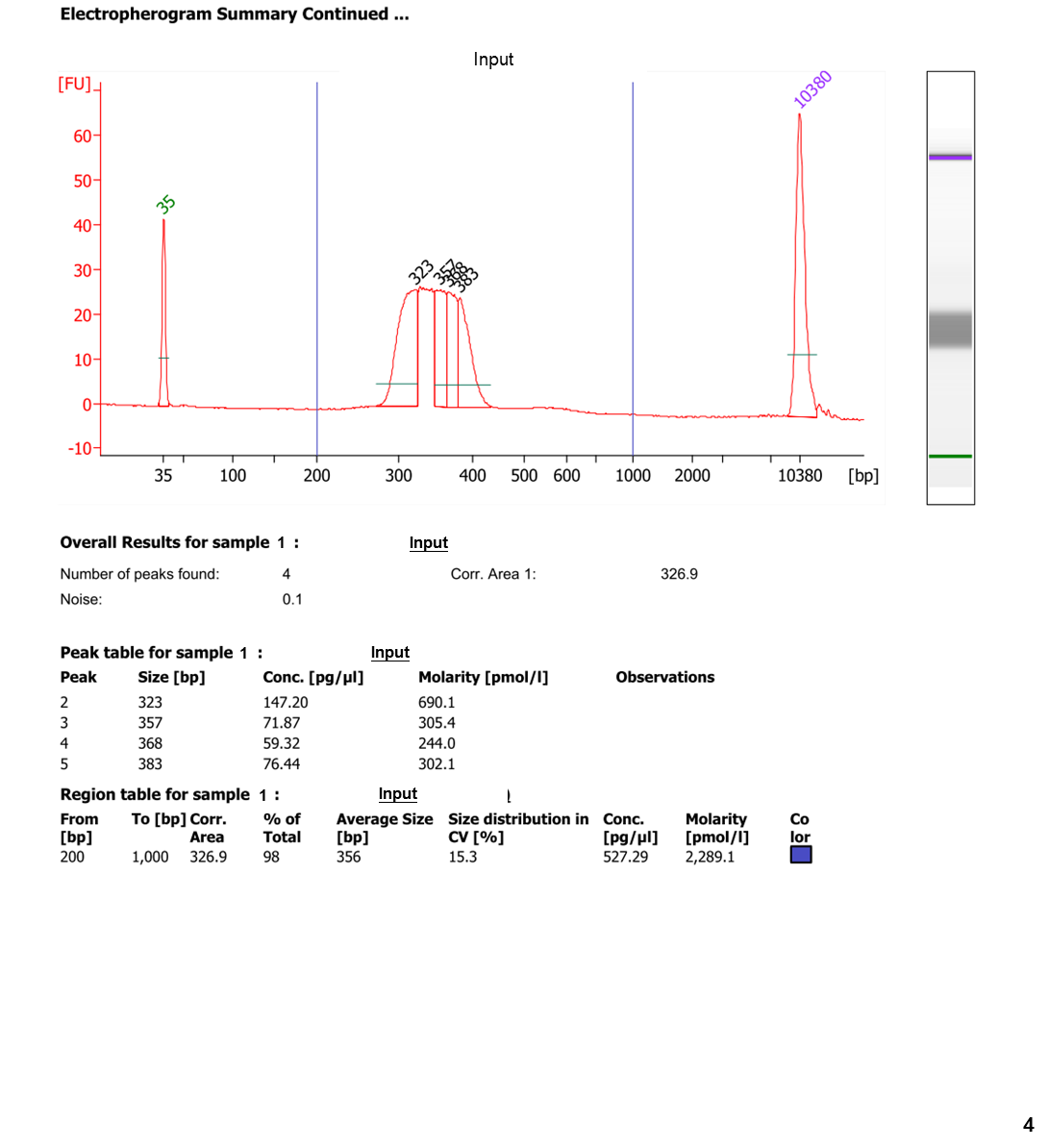
**

**e
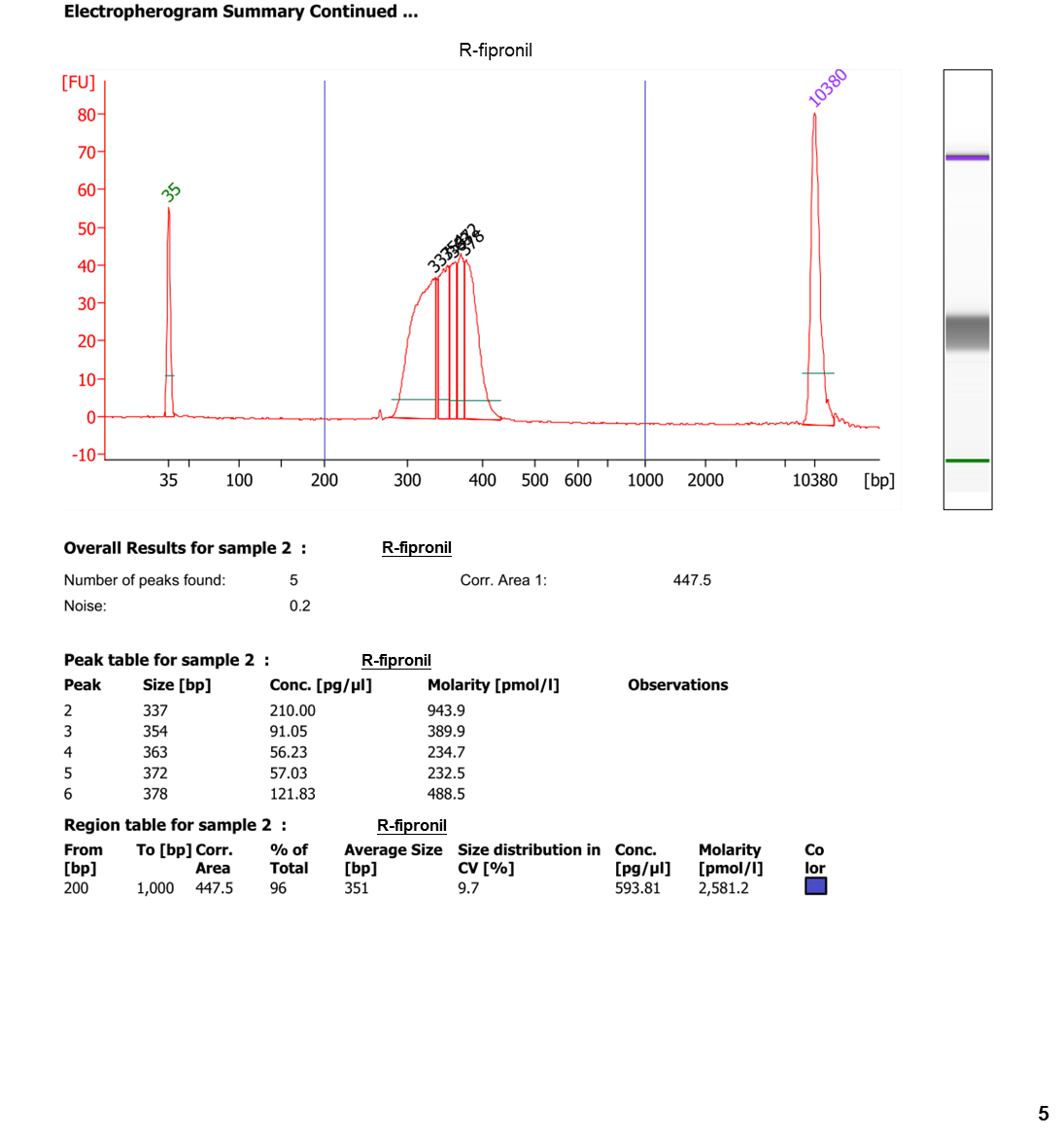
**

**f
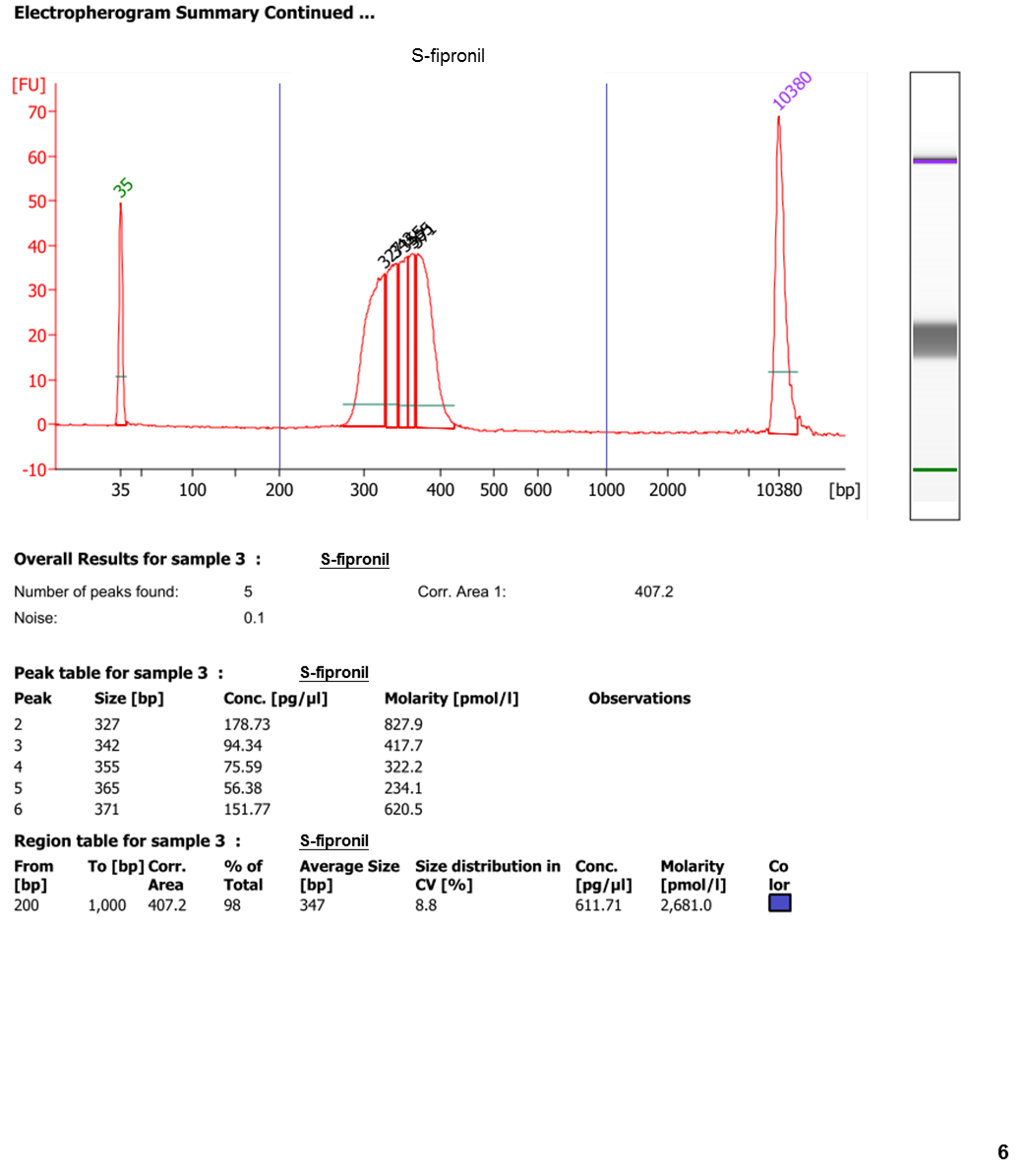
**

**g**


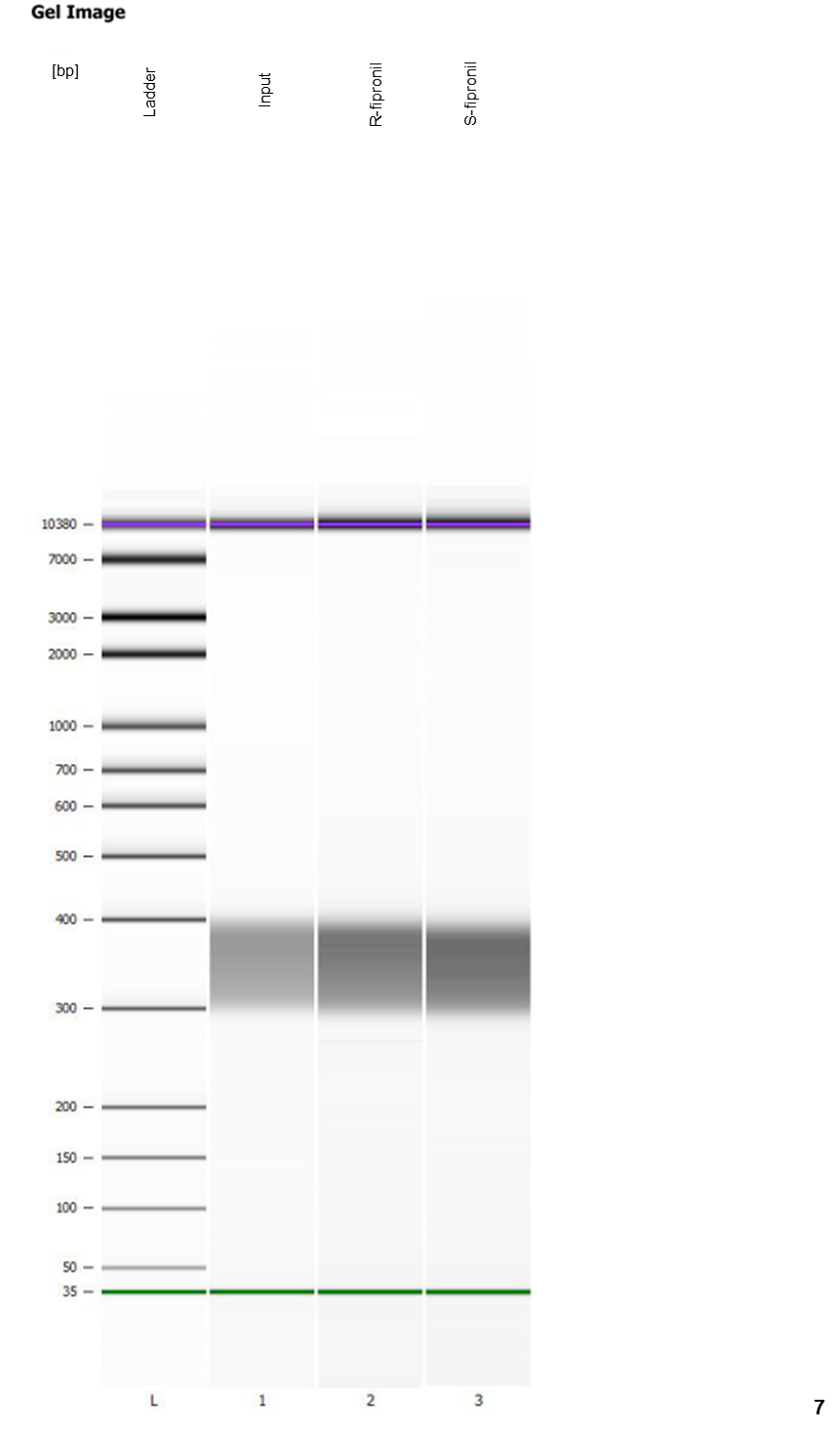


**Supporting information, Figure S3** Distribution of DNA methylation peaks in different genomic regions.


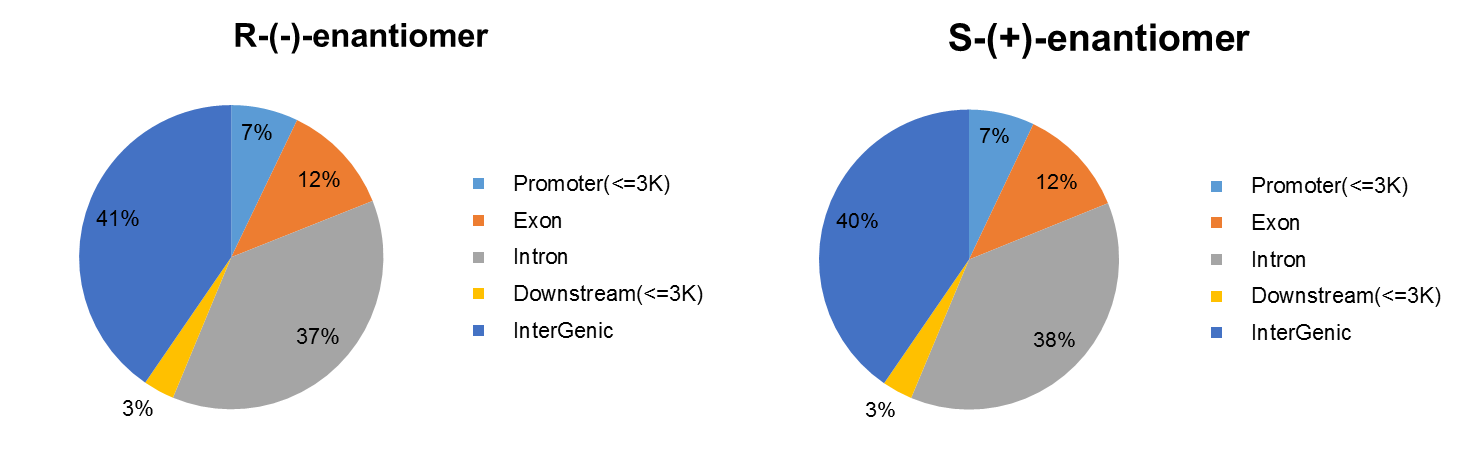

Supplement: Supplementary file 1 — Supporting document. [file 41598_2017_2255_MOESM1_ESM.doc]
